# Treatment needs and impact of oral health screening of athletes with intellectual disability in Belgium

**DOI:** 10.1186/s12903-015-0157-9

**Published:** 2015-12-30

**Authors:** C Fernandez, D Declerck, M Dedecker, L Marks

**Affiliations:** Centre of Special care in dentistry, PAECOMEDIS, Ghent University Hospital, De Pintelaan 185, 9000 Ghent, Belgium; KU Leuven, Population studies in Oral Health, Department Oral Health Sciences, Leuven, Belgium; WTB- VVT – Flemish taskforce Dentistry for Special Needs, Brussels, Belgium

**Keywords:** Oral health, Dentistry, Special olympics, Belgium, Special care

## Abstract

**Background:**

Special Olympics Special Smiles (SOSS) is an initiative created for oral health data collection and education in oral hygiene for athletes with an intellectual disability. The aims of this study were to evaluate treatment needs of participants of Special Olympics in Belgium 2013 in comparison with those from 2008 and to assess the impact of screening and referral within the SOSS in a group of athletes who participated in two consecutive events, 2012 and 2013.

**Methods:**

Data were collected following a standardized protocol developed by the U.S. Centres for Disease Control and Prevention, Division of Oral Health. Oral hygiene habits, treatment urgency and reports of oral pain, gingival signs, sealants, untreated caries, missing and filled teeth were recorded. Data analysis of data from 2013 consisted in descriptive statistics followed by the analysis of the data by univariable and multivariable logistic regression. This data was compared with data from 2008 published by Leroy et al., 2012 using Chi square tests. Data from athletes who participated in both Special Olympics events (2012 and 2013) were compared using Exact McNemar's test and Chi-square test for homogeneity of proportions. The level of significance for all tests was set at a p-value < 0.05.

**Results:**

A total of 627 athletes with intellectual disability participated to the SOSS program in 2013, while 132 athletes met the inclusion criteria of being a participant at both SO Belgium 2012 and 2013. The prevalence of gingival signs was 44.3 % in 2013, slightly higher than in 2008 (42.4 %). The burden of untreated decay affected 27.1 % of the population showing a net increase in comparison to 2008 (20.9 %). McNemar's test and Chi-square test revealed that there were no statistically significant differences in the proportions of all compared parameters between 2012 and 2013.

**Conclusion:**

Special Olympics results from 2013 indicate a considerable unmet treatment need among Belgian Special Olympics Athletes, persistent from 2008 to 2013. Moreover, SO intervention had no impact in the oral health of athletes who participated in 2012 and 2013 events. Continuous efforts for preventive and restorative oral health care are needed for this population.

## Background

Oral health is an integral part of overall well being that influences the quality of life and has a strong impact on general health [1, 2]. Surveys have shown that rates of oral disease and specifically tooth decay and tooth loss are declining worldwide, as a result of variations in population structure and development of new therapeutic approaches [3]. Between countries, differences in health situation are mostly related to differences in healthcare systems, whereas within countries variations are strongly linked to individual characteristics. Overall health is poorer in socio-economically disadvantaged groups, minority groups, individuals with chronic diseases and persons with disabilities [3].

The Health Monitoring Unit of the European Union, acknowledging the gap in evidence on health of the intellectually disabled population, launched the Pomona project in 2005 [4]. In this project, several health indicators specific to people with intellectual disabilities were developed (Pomona I) and tested in 14 European countries (Pomona II) to gather information on lifestyle, health status, behaviour and access to health care. The objective was to increase understanding of the determinants of health among people with intellectual disabilities. It was concluded that people with intellectual disabilities experience poorer health and poorer access to optimal health care. Moreover, they are more likely to incur secondary health conditions and report increased morbidity [4].

From the Pomona II health survey, information was gathered from interviews from a sample of 1,269 adults with mental disabilities from 14 European countries, 21 % reported having pain in their mouth (Belgium 19 %); in 75 % of those cases the pain was in the teeth, in remaining cases the pain was in other areas of the mouth [4].

The health status of a population is directly linked with its health care system; in this regard the Belgian system is characterized by mandatory health insurance and free choice of care providers [5]. The oral healthcare, in particular, is partially included in the health insurance and delivered almost exclusively by private practitioners [6]. For certain treatments, the amount of reimbursed money is determined by age [7–9]. For instance, reimbursement is 100 % for the whole population ‘under 18 years old’, except orthodontic treatment. For adults, on the other hand, the system covers 75–79 % of the national fees for preventive and restorative care, removable dentures and minor oral surgery. However, disabled people over 18 years, are entitled to a 100 % reimbursement for restorative oral care (except fixed prostheses and implants), prophylactic cleanings, extractions and debridement procedures [10,11].

Special Olympics is an international sports organization for children and adults with intellectual disabilities that includes training and competitions for more than 4.2 million athletes in more than 170 countries.

The Special Olympics Healthy Athletes program is an initiative that started in the United States in 1996, with the principal objective of helping athletes who participate in the Special Olympics games to improve their health and fitness. Special Olympics Special Smiles (SOSS) is the oral health component of the Healthy Athletes program. The main goal of SOSS is to collect standardized and region-specific data on oral health in order to improve access and delivery of dental care for people with special needs. The lack of reliable international surveys on the oral health of people with an intellectual disability makes this program a unique opportunity to conduct a large number of standardized examinations, interviews and education in this population [12, 13].

Until now the oral health of people with disabilities has been reported to be poor [8, 14–18]. As an illustration, a systematic review published in 2010 studied the differences in oral health between general population and people with intellectual disabilities. From 27 reviewed studies it was concluded that people with disabilities have worse oral hygiene and higher plaque levels, more severe gingivitis and periodontitis, more untreated dental disease and higher numbers of extracted teeth [15].

In 2012, Leroy published an article on the oral health status of Special Olympics athletes in Belgium based on the results obtained in 2008. The most relevant findings were the prevalence of gingival signs of inflammation in 44 % of the athletes, the presence of untreated decay in 22 % and urgent treatment need in 12 %. Hence, it was concluded that the need of oral health care was huge [14].

Although a number of papers have been published including SOSS analyses from all over the world, no analyses have been reported regarding the impact of treatment referral during the program, which makes the current paper unique.

The aim of this study is two-fold. First, to evaluate trends in oral health condition and treatment needs of participants of SO in Belgium, by comparing oral health parameters recorded in 2008 and 2013. Second, this work aims to assess the impact of screening and referral within the SOSS on the oral health outcome of individual athletes who participated in the Special Olympics Belgium in two consecutive years (2012 and 2013).

## Methods

Oral health data were collected through interviews and oral examinations of athletes participating in the annual Special Olympics event held in Belgium, both in 2012 and in 2013. They were invited to the “Special Olympics Special Smiles” site where they could have their teeth examined on a voluntary basis. Consent was obtained before the event from the athlete and a parent or guardian depending on the level of comprehension of the athlete. The Joint Ethical Committee of the Ghent University Hospital approved the study as 2013/816. This article includes also data collected in the SO 2008 Belgian event where identical methods were used [14].

The procedure consisted of registration of demographic data (age, gender and date of birth), oral health screening, and education in oral hygiene techniques. Standardized data collection forms were used to record the following information: edentulism, untreated decay, filled or missing teeth, sealants, tooth injury, fluorosis and signs of gingival disease [13]. The standardized examination protocol developed for SOSS by the U.S. Centers for Disease Control and Prevention, Division of Oral Health [13], was strictly followed. This protocol prescribes a specific sequence and includes a visual assessment of each condition in a separate cycle, independent of others. If two conditions are present in a tooth both are marked and third molars or partially erupted teeth are not considered.

For evaluation of brushing habits the athlete was asked how often he/she cleaned his/her mouth. Edentulism was recorded as the complete absence of teeth or root remnants. Untreated decay was scored in both primary and permanent dentition (except third molars) when at least one area of cavitation that would accommodate a 0.5 mm-diameter (or larger) bur was detected. Any dental restorative work done exclusively as a response to decay, was coded as ‘filled tooth’ and ‘missing tooth’ was recorded if a tooth was not present at the time of the exam (with exception of premolars and wisdom teeth). Unerupted teeth were not counted as missing.

For scoring the presence of dental signs of trauma, only maxillary and mandibular central and lateral incisors in the permanent dentition were considered. This score was attributed when a tooth was either absent, fractured or discolored indicating loss of vitality. The presence of sealants was recorded when material placed as a preventive measure, covered the pits and fissures of the occlusal surface(s) of first and/or second permanent molars.

Small, diffuse, opaque, paper-white areas and/or presence of brown stains and pitting scattered over at least 25 percent of the buccal surface of maxillary front teeth (canine to canine) were considered signs of fluorosis. Free or attached gingival margins or papillae moderately red or showing significant deviations from normal contour or texture on three or more teeth within the same area, were recorded as a sign of gingival disease.

At the end of the oral inspection, treatment urgency was assessed based upon clinical findings. If there was no pain complaint, no untreated decay or dental injuries and no signs of gingival disease the athlete was recorded for maintenance follow-up. In case of absence of pain, presence of decay but not involving the pulp, defective fillings and gingival problems without abscess formation, the athlete was referred for non-urgent treatment. When there was pain inside the mouth, teeth with possible pulpal involvement, broken or missing fillings with decay or periodontal abscess formation, the participant was referred for urgent treatment. Each athlete received a letter with treatment recommendation.

The procedure was concluded with an individual oral health instruction performed considering the athlete’s capacity of comprehension and response.

Data collection was performed by dentists recruited from university dental schools and dental professional organizations. They performed the oral screening, for which they were previously trained and standarized according to the Training Manual for Standardized Oral Health Screening [13]. The materials used for the examination were flashlights, gloves and disposable plastic mirrors.

All data collected were entered into an Excel worksheet and transferred to an SPSS data file. Data analysis of data from 2013 consisted in descriptive statistics followed by the data comparison with data from 2008 with Chi Square tests and the analysis of the data of 2013 by univariable and multivariable logistic regression with oral hygiene frequency, presence of untreated decay, gingival signs of inflammation, dental injury, sealants and treatment urgency as explanatory variables to estimate crude and adjusted odds ratios for their explanatory capacity of untreated decay and gingival signs of disease.

The data from athletes who participated in both Special Olympics Belgium National events (2012 and 2013) were compared using Exact McNemar's test and Chi-square test for homogeneity of proportions. The level of significance for all tests was set at a p-value < 0.05. Bonferroni correction was used for multiple comparisons according to number of comparisons conducted.

## Results

A total of 627 athletes with intellectual disability participated to the SOSS program in 2013. The participants were mainly adult with 11.1 % of athletes under 18 years old, 15.9 % between 18 and 25 years, and 73 % had 26 and more years. Reported age groups were selected to be comparable with published international multi-center surveys [19]. Mean age was 33.02 (with a SD of 13.01), minimum age of 5 and maximum of 68 years.

Gender distribution showed 229 females (36.5 %) and 398 males (63.5 %). Table 1 presents demographical characteristics, reported oral hygiene habits and clinical findings of participants of the 2013 survey, completed with corresponding data collected in the 2008 survey. For more detailed information on the latter sample we refer to Leroy et al. 2012 [14].

**Table 1:** Demographic characteristics, reported oral hygiene habits and clinical findings in participants of 2008 and 2013 surveys

		*2008 (n = 687)		2013 (n = 627)	
Variables		*n*	%	*n*	%
Age	Mean	33y	SD:13	33.02y	SD: 13.02
	Range	9-80		5-68	
Gender	Males	408	60.1	398	63.5
	Females	271	39.9	229	36.5
Oral hygiene	Once or more a day	581	84.6	497	79.3
Oral hygiene habits	2 - 6 times a week	41	6.0	58	9.3
	Once a week	10	1.4	17	2.7
	Less than once a week	6	0.9	9	1.4
	Not sure	17	2.4	24	3.8
	No data	32	4.7	22	3.5
Edentulism	No	660	96.1	609	97.1
	Yes	27	3.9	18	2.9
	No data	0	0	0	0
Signs of Gingivitis	No	363	52.8	317	50.6
	Yes	291	42.4	278	44.3
	No data	33	4.8	32	5.1
Untreated decay	No	502	73.1	416	66.3
	Yes	144	20.9	170	27.1
	No data	41	6.0	41	6.5
Filled teeth	No	145	21.1	174	27.8
	Yes	503	73.2	424	67.6
	No data	39	5.7	29	4.6
Missing teeth	No	N	N	291	46.5
	Yes	N	N	311	49.7
	No data			24	3.8
Dental Injury	No	572	83.3	521	83.1
	Yes	82	11.9	78	12.4
	No data	33	4.8	28	4.5
Sealants	No	607	88.4	530	84.5
	Yes	41	5.9	60	9.6
	No data	39	5.7	36	5.6
Fluorosis	No	N	N	589	94.0
	Yes	N	N	4	0.6
	No data			34	5.4
Treatment Urgency	Maintenance	384	55.9	354	56.5
	Non-urgent	183	26.6	130	20.7
	Urgent	84	12.2	74	11.1
	No data	36	5.3	69	11

*2008 data derived from Leroy et al., 2012 [14]

*N* = no information available

### Descriptive results from 2008 and 2013 surveys

Both samples were similar in size and age distribution, with a mean age of 33 years in both groups. Between both surveys, there was a decrease in number of athletes who reported to clean their mouth at least once a day, from 84.6 % in 2008 to 79.3 % in 2013 (p < 0.001). The overall prevalence of gingival signs was not different in 2013 and 2008 (44.3 % and 42.4 %) (*p* = 0.43). The burden of untreated decay affected 27.1 % of the study population in 2013, showing a net increase in comparison to 2008 (20.9 %)(p < 0.01); the prevalence of sealants increased from 5.9 % (2008) to 9.6 % (2013) (*p* < 0.01).

### Explanatory variables

Univariable logistic regressions showed that gender was not related to the variables oral hygiene habits, presence of untreated decay, gingival signs of inflammation, dental injury, sealants and treatment urgency.

Gingival inflammation was significantly related with age, presence of untreated decay, treatment urgency and reported oral hygiene habits (Table 2). Athletes under 18 years old had a statistically significant smaller chance for having gingivitis than those older than 26 years (OR: 0.41; 95 % CI: 0.20 to 0.84). A higher chance of presenting gingival signs of disease was found among athletes who received non-urgent treatment recommendation (OR: 3.86; 95 % CI: 2.17 to 6.85) than maintenance.

**Table 2:** Effects of categorical explanatory variables on gingival signs of inflammation

	Univariable			Multivariable		
Gingival signs^a^	OR	*p*	95 % CI for OR	OR	*P*	95 % CI for OR
Gender						
Female vs. male	1.09	0.60	0.78-1.53	1.10	0.62	0.76-1.58
Age						
<18 vs. 26 or more	0.30	<0.001	0.17-0.55	0.41	0.02	0.20-0.84
18–25 vs. 26 or more	0.77	0.24	0.49-1.20	1.09	0.71	0.68-1.75
Untreated decay	0.52	<0.001	0.36-0.75	0.97	0.95	0.34-2.72
Oral hygiene habits^b^						
2-6/ week vs. ≥ 1 /day	1.71	0.07	0.97-3.02	2.46	0.34	0.39-15.36
Treatment Recommendation^b^						
Urgent vs. Maintenance	2.37	0.001	1.40-4.00	2.54	0.12	1.23-5.26
Non-Urgent vs. Maintenance	3.30	<0.001	2.13-5.11	3.86	0.001	2.17-6.85

^a^The reference category is: Gingival signs of disease (yes)

^b^Only significant values are shown

Untreated decay was related with the frequency of oral hygiene habits (Table 3). Athletes who reported to clean their mouth 2–6 times a week presented higher odds of having untreated decay than those who clean their mouths once or more a day (OR: 1.82; 95 % CI: 1.00 to 3.31). However, it was less likely to be found in athletes younger than 18 years (OR: 0.28; 95 % CI: 0.13 to 0.61) and between 18–25 years old (OR: 0.42; 95 % CI: 0.24 to 0.75) when comparing them with older athletes.

**Table 3:** Effects of categorical explanatory variables on untreated decay

	Univariable			Multivariable		
Categorical predictor	OR	*p*	95 % CI for OR	OR	*P*	95 % CI for OR
Gender						
Female vs. male	1.03	0.90	0.70-1.50	1.04	0.83	0.70-1.55
Age^a^						
<18 vs. 26 or more	0.28	0.001	0.13-0.61	0.28	0.001	0.13-0.61
18–25 vs. 26 or more	0.42	0.003	0.24-0.74	0.42	0.003	0.24-0.75
Oral hygiene habits^a^						
<1 / week vs. ≥ 1 /day	2.12	0.27	0.56-8.03	1.81	0.39	0.47-7.01
1/ week vs. ≥ 1 /day	1.59	0.38	0.57-4.47	1.46	0.50	0.49-4.38
2-6/ week vs. ≥ 1 /day	1.77	0.05	0.99-3.15	1.82	0.05	1.00-3.31

^a^Only significant values are shown

Athletes under 18 years old (OR: 3.13; 95 % CI: 1.50 to 6.53), or between 18 and 25 years old (OR: 3.15; 95 % CI: 1.66 to 5.98), presented a significantly higher odds of having sealed teeth. Untreated decay, however, was related with absence of sealed teeth (OR: 0.45; 95 % CI: 0.20 to 0.94) (Table 4).

**Table 4:** Effect of categorical explanatory variables on presence of sealants

Categorical predictor	OR	*p*	95 % CI for OR
Gender			
Female vs. male	1.31	0.37	0.73-2.35
Age			
<18 vs. 26 or more	3.13	0.002	1.50-6.53
18–25 vs. 26 or more	3.15	<0.001	1.66-5.97
Untreated decay	0.45	0.03	0.20-0.94

Only variables with significant values are shown

### Changes between 2012 and 2013

A total of 132 athletes, who met the inclusion criteria of being a participant on both SO Belgium 2012 and 2013, formed the population for this part of the study. The age and gender distribution was very similar to that in the general sample with 8.3 % athletes under 18 years old, 19.7 % between 18 and 25 years, and 72 % 26 and more years. There were 52 females (39.4 %) and 80 males (60.6 %). Mean age was 33.16 (with a SD of 13.01), minimum age of 10 and maximum of 61 years.

**Fig. 1:**
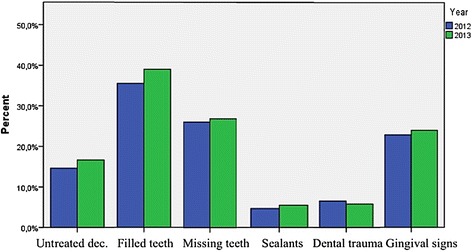
Distribution of selected oral health parameters in athletes participating in both events 2012 and 2013 (*n* = 132)

Exact McNemar's test and Chi-square tests for homogeneity of proportions determined that there was no statistically significant difference in the proportion of untreated decay, sealants, gingival signs of disease, dental injury, restored or missing teeth between athletes participating in both SO events 2012 and 2013 (Fig. 1). Moreover, no statistical differences were found regarding reported oral hygiene habits and treatment urgency.

## Discussion

Oral cleaning habits are affected by an individual’s cognitive and motor skills. The level of intellectual disability may limit these skills compromising the ability to perform personal oral hygiene so that supervision and/or assistance of a caregiver becomes a necessity. In addition, poor lip closure is a prevalent feature among individuals with intellectual disability that affects the natural cleansing of the oral cavity [20, 21]. According to the protocol, in this study the question ‘How often do you clean your mouth?’ was asked rather than, ‘How often do you brush your teeth?’ because the idea is to assess the frequency of oral hygiene effort without consideration of the specific devices used [13].

The most worrying findings were the high prevalence of gingivitis signs, untreated decay and urgent treatment recommendations. Reported oral cleaning frequency was significantly related with the presence of gingival signs of disease. The majority of the athletes (79.3 %), reported to brush their teeth at least once a day, even though this self reported data could have been influenced by previous knowledge of the ideal frequency of oral cleaning. Effectiveness in plaque removal, essential for oral health, was not measured, and an inadequate brushing technique could explain the high prevalence of gingival signs (44.3 %) [22]. Athletes with Down syndrome, approximately 13 % of SO athletes according to Special Olympics database, are expected to have a higher prevalence of gingivitis, considering the higher level of specific sub-gingival bacterial species and their impaired immunologic responses [16, 23, 24]. The results obtained in the present study are comparable with those from other studies based on samples from Special Olympics participants in the United States (2001; 40.1 %), Puerto Rico (42 %) and Venezuela (45 %) in 2013, but lower than in New Jersey (1996; 60 %), UK (2005; 63 %), Italy (2009; 60 %) and Mexico (2013; 52 %) [16, 24–27].

The burden of untreated decay affected more than one fourth of the participants, only considering lesions with a diameter of 0.5 mm and without radiographical support for its detection. For this reason, the actual prevalence of decay may be even higher. This parameter was also strongly related with treatment urgency, as could be expected from the protocol guidelines for treatment recommendations, and less prevalent in athletes with gingival signs [13]. The prevalence of untreated caries, reported in studies using the same standardized protocol, showed great variability with figures ranging between 19 % and 79 % [13, 17, 25, 28].

Athletes ‘over 26 years old’ showed higher odds of presenting gingival signs of disease and less evidence of preventive care treatments like sealants. However, not much evidence has been published on the prevalence of fissure sealants in adults, our results are in agreement with an American review, released in 1996 by the Third National Health and Nutrition Examination Survey (NHANES III). This review indicated that 2 % of 25 to 39 years old adults had evidence of dental sealants [29, 30]. In Belgium, the Oral Health Data Registration & Evaluation System (OHDRES) ran between October 2009 and December 2010. This survey, showed a prevalence of fissure sealants of 4,7 % in adults between 25 and 34 years old [31].

Prevalence of edentulism was 2,9 % but it has to be noted that the mean age of our athletes was 33 therefore this parameter is not too representative. On the other hand, the prevalence of dental injury (12.4 %) was expected, firstly because the prevalence of dental trauma in the general population ranges from 2 % to 33 % [32, 33] and also since it is known that self-inflicted traumatic oral injuries are common in intellectually disabled persons, rates of 2 to 33 % have been reported [34], having a detrimental influence on their functional and social performance. Individual characteristics may explain this tendency; poor lip closure, slow response to environmental obstacles, oral pathologic reflexes and a large overjet of maxillary incisors.

Following the Special Smiles protocol recommendation for urgent treatment was issued to 11.1 % of the participants who presented oral pain or possible pulpal involvement, a proportion comparable with results obtained in Italy and in the U.S. but much lower than in other countries [24, 25, 35, 36].

According to the Belgian National Institute for Health and Disability Insurance (NIHDI) the health care expenditure was over 35 billion Euro in 2008. Health expenditure has increased over the last decade [37]. The expenditure in dentistry was 3.2 % of the general healthcare expenditure in 2013 and its distribution between the different areas of dental care has been mostly constant over the last decade. From the budget, 52 % goes to "Conservative treatments" and this amount slightly decreased over the years. The section "Preventive care" (13.7 %) had a particularly strong growth since 2000 and is likely to become an important second section [6, 10, 38]. When comparing results of this study with the report from SO Belgium in 2008 (Table 1), there is no evidence of a change in oral health parameters in Belgian Special Olympics athletes over the last five years. The increase in sealants and the decrease in need of treatment urgency evidences preventive and restorative oral care. Notwithstanding, gingival signs of disease, filled teeth and untreated decay suggest no improvements in oral disease and no broad variations in the need of education on oral health care.

Overall, the effect of the annual SO oral health screening including individual oral health instructions was very limited and did not yield statistical significant changes when evaluating athletes one year later. The question remains of whether athletes or they caregivers did not understand that there were conditions that needed attention. Although these results might be related to the limited sample size and short follow-up, oral health needs remained considerable and this could be related with a need for more intensive instruction, enhanced dentist training and/or improved facilities. This affects people with severe intellectual disabilities to a higher degree, because they are more likely to require stabilization, sedation or general anaesthesia, for which dentists need additional training.

The high need for preventive and restorative oral health care among this population persisted. Clearly, from a one-time-a-year intervention in the scope of the Special Olympics events, improvements cannot be expected unless they are complemented with other interventions of oral health promotion and education of athletes, family and caregivers. Moreover, dental professionals should be more aware of the oral health needs of this population and more prepared to face them.

Belgium belongs to the EURO A group in the WHO classification for Burden of Disease 2000 [39], the group with the best health situation among European countries, considering child and adult mortality. Its expenditure in health is one of the highest in Europe, the health care insurance system is mandatory and claims to cover almost the whole population. The oral health needs of the Belgian disabled population, however, are huge. Although there is at least 90 % reimbursement of treatment costs and several centres where Special Care Dentistry is offered are available, other factors seem to limit the access to oral health care. From all this, it is clear that there are specific barriers that affect the access of this population to oral health care which need to be further studied.

The use of a globally accepted standardized SOSS protocol enables comparisons between available and future data obtained with the same methodology [14, 25, 27, 28, 35, 36, 40]. Results, nonetheless, must be interpreted with caution. The study results cannot be extrapolated to the whole population with intellectual disability, because study participants were athletes, participating in Special Olympics events and therefore a relatively young, well supported and high-functioning subgroup of this population [17, 24]. In addition, a convenience sample was used, recruited on-site during the Special Olympics event. The size of the sample used for the assessment of changes in treatment needs of athletes who participated both in 2012 and 2013 Special Olympics events was relatively small (n = 132). This implies that future studies with a larger sample and longer follow-up period could reach stronger conclusions on the impact of the Special Smiles intervention. Further research including data on type and severity of disability and the use of specific index for caries and periodontal disease, such as International Caries Detection and Assessment System (ICDAS), dmft and Community Periodontal Index (CPI), would benefit the comparison to other studies in literature.

Also, the risk of misclassification by over- or under-reporting of parameters that were asked to the athletes, such as oral hygiene habits, should not be disregarded. This could have introduced bias in data collection but there are no means in this study to determine it or measure it [14, 26].

## Conclusion

The general results of the Special Olympics 2013 indicate a considerable unmet treatment need among Belgian Special Olympics Athletes, persistent from 2008 to 2013. Additionally, this study did not find any evidence of impact of the oral health screening among the Belgian Special Smiles population.

Even though the sample is not representative of the whole population with intellectual disabilities the results support the need for increased promotion of health, prevention of disease and education, as well as preventive and restorative treatment.
